# Enhancement of membrane vesicle production by disrupting the *degP* gene in *Meiothermus ruber* H328

**DOI:** 10.1186/s13568-021-01328-z

**Published:** 2021-12-15

**Authors:** Yuki Asano, Manato Onishi, Kaito Nishi, Kazunori Kawasaki, Kunihiko Watanabe

**Affiliations:** 1 Division of Applied Life Sciences, Graduate School of Life and Environmental Sciences, Kyoto Prefectural University, Shimogamo, Sakyo, Kyoto, 606-8522 Japan; 2 Health Research Institute, National Institute of Advanced Industrial Science and Technology, Ikeda, Osaka 563-8577 Japan

**Keywords:** Membrane vesicle, Thermophile, *degP*, Homologous recombination, Gram-negative

## Abstract

The phenomenon of membrane vesicle (MV) production is known to be common to all bacterial cells. Although MVs are expected to be employed in a variety of applications, improving MV productivity is essential for applications. Since the deletion of the *degP* gene, a periplasmic dual-function protease and chaperone, in *Escherichia coli* has successfully improved MV production capacity, we tried to enhance MV productivity in the thermophilic *M. rube*r H328 by deleting the *degP* gene. One gene (*mrH_0331*) was selected for *degP* gene from the H328 genome and we constructed the mutant strain ∆*degP* by deleting the *degP* gene of the H328 strain that was replaced with the *htk* gene showing thermophilic kanamaycin resistance by homologous recombination. The mutant strain ∆*degP* exhibited smooth growth but a lower level of turbidity at 60 °C although there was no difference in growth at 55 °C between the wild strain and the mutant strain. Finally, we have confirmed that incubation at 60 °C increases MV in the mutant strain ∆*degP* strain about fivefold by using two fluorescent dyes, DiI and FM4-64, which is followed by TEM analysis. The deletion of the *degP* gene presumably causes an increase in denatured proteins at 60 °C, leading to enhanced MV production. Meanwhile, the S-layer protein included in the outer membrane of the H328 strain increased in the MV fraction prepared from the mutant cells incubated at 60 °C. This indicates that this method is effective for MV production and that *degP* deletion enhances it in strain H328.

**Supplementary Information:**

The online version contains supplementary material available at 10.1186/s13568-021-01328-z.

## Introduction

*Meiothermus rube*r H328 is a thermophilic bacterial strain (optimum growing temperature: 50–60 °C) that was isolated at Arima Hot Spring, Kobe, Japan (Matsui et al. 2009). This strain was characterized by the remarkable ability to decompose native chicken feathers in cultivation. While this strain enables the degradation of such industrial waste from the poultry industry, the microbe has also been found to significantly produce membrane vesicles (MVs) (Yamaoka et al. 2014). The phenomenon of MV production is known to be common to all bacterial cells (Kulp and Kuehn 2010; Kim et al. 2015; Gill et al. 2019; Toyofuku et al. 2019). Although MVs are expected to be employed in a variety of applications, improving MV productivity is essential for applications (Schwechheimer and Kuehn 2013; Turner et al. 2015; Roier et al. 2016; Watanabe 2016; Kao and Papoutsakis 2019). While it has been argued that the addition of detergents and oxygen stress effectively enhance MV production (Holst et al. 2009; Gerritzen et al. 2018), the deletion of a limited number of genes has been found to effectively enhanced MV production (Bernadac et al. 1998; McBroom et al. 2006). In particular, the deletion of the *degP* gene, a periplasmic dual-function protease and chaperone, in *Escherichia coli* has successfully improved MV production capacity (Schwechheimer and Kuehn 2013). On the other hand, the protein DegP was initially annotated as a protease named HtrA and was later found to have chaperone functions for denatured proteins as a heat shock protein (Lipinska et al. 1990). Given this background, we tried to enhance MV productivity in the thermophilic *M. rube*r H328 by deleting the *degP* gene since the genomic information of the strain was elucidated and available (Inada and Watanabe 2013).

Here, we identified the most potent candidate of the *degP* gene in the genome of *M. ruber* H328 and disrupted the gene by homologous recombination to construct a mutant of the H328 deleting *degP* (called mutant strain as *∆degP*). Finally, by analysis employing two fluorescent reagents, we demonstrated that the mutant strain *∆degP* is capable of enhancing MV production when incubated at 60 °C, a heat stress condition.

## Materials and methods

### Strains, plasmids, and cultivation

*M. ruber* H328 was described in our previous report (Matsui et al. 2009). The strain number FERM AP-20950 was provided in an earlier AMB article (Matsui et al. 2009). The wild and mutant strains of *M. ruber* H328 were cultivated at 55, 57, or 60 °C in YS medium (0.5% (w/v) yeast extract, 0.5% (w/v) sucrose pH 8.0) (Matsui et al. 2009). The preculture was aerobically carried out in 5 mL of YS medium at an appropriate temperature, 2% (v/v) of the preculture was transferred to fresh YS medium (5 mL for homologous recombination or 200 mL for cell growth and MV analyses) and the culture was incubated at the corresponding temperatures maximally for 144 h. The *Escherichia coli* strain used for DNA manipulations was DH5α. The plasmid pUC18-*htk* including a thermophilic kanamaycin-resistant gene and its transcriptional promoter was purchased from Riken BioResource Research Center (Tsukuba, Japan) (Hoseki et al. 1999). Cultivation of *M. ruber* H328 and *E. coli* DH5α followed the previous method (Yamamoto et al. 2020).

### Gene identification of *degP* candidates in *M. ruber* H328

The search for *degP* candidates of strain H328 was carried out by using the DNA sequence of the *degP* gene (accession number: NP_414703) of *E. coli* MG1655 and the genomic information of strain H328 (genome assembly: GCA_000346125.2); their homology was compared by use of BlastP (http://blast.ncbi.nlm.nih.gov/Blast.cgi). Localization was accomplished by use of PSORTb (http://www.psort.org/psortb/) and CELLO (http://cello.life.nctu.edu.tw/), and the signal peptide for secretion was determined by using SignalP (http://www.cbs.dtu.dk/services/SignalP-4.0/), respectively.

### Plasmid constructions and homologous recombination of *M. ruber* H328

**Fig. 1:**
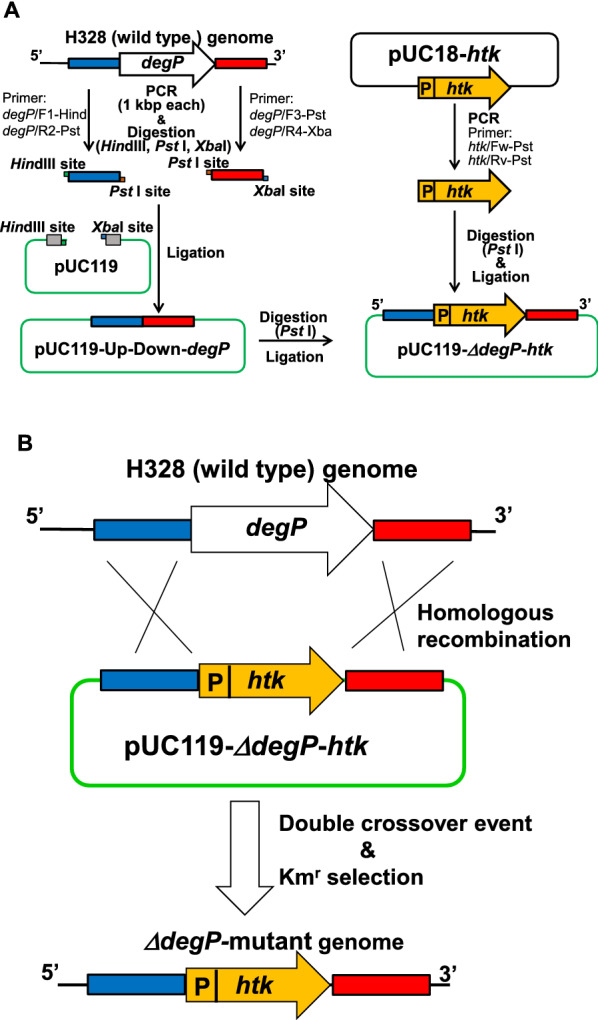
Plasmid constructions (**A**) and homologous recombination (**B**) of *M. ruber* H328. (** A**) A hybrid plasmid pUC119-*∆degP*-*htk* for homologous recombination was constructed by using the vector plasmid pUC119 and three DNA fragments (1.0 kbp each) including the 5’-flanking and 3’-flanking regions of the *degP* gene (*mrH_0331*) in the chromosomal DNA of strain H328 and a thermophilic kanamycin-resistant gene (*htk*) from the plasmid pUC18-*htk*, respectively. DNA fragments were obtained by PCR by using a pair of primers listed in Additional file 1: Table S1 in the reaction condition as described in the Materials and methods section and their digestion and ligation followed the procedure recommended by the vendor. (**B**) Homologous recombination was carried out as described in the Materials and methods section, and the knockout of the *degP* gene was confirmed by PCR using a pair of primers (*degP*/F1-Hind and *htk*/Rv-Pst or *htk*/Fw-Pst and *degP*/R4-Xba)

To obtain a *degP*-deleting mutant of strain H328, homologous recombination was carried out on strain H328. To complete the homologous recombination, a hybrid plasmid pUC119-*∆degP*-*htk* was constructed by using vector plasmid pUC119 and three DNA fragments (1.0 kbp each), including the 5’-flanking and 3’-flanking regions of the *degP* gene (*mrH_0331*) from the chromosomal DNA of strain H328 and a thermophilic kanamycin-resistant gene (*htk*) from the plasmid pUC18-*htk*, respectively (Fig. 1). The DNA fragments were obtained by PCR, using a pair of primers listed in Additional file 1: Table S1. The PCR mixture (50 µL) contained 10 ng of template DNA (chromosomal DNA of strain H328) and 10 pmol of each primer with thermostable DNA polymerase KOD FX (Toyobo, Osaka, Japan). The reaction program was as follows: 30 cycles of denaturation (1 min at 98 °C), annealing (30 s at an appropriate temperature), and extension (2 min at 68 °C). PCR products were purified by using a QIA purification kit (Qiagen, Hilden, Germany) and digested by appropriate restriction enzymes (Fig. 1A). After ligating those DNA fragments to vector plasmid pUC119, the construction of the plasmid was confirmed by agarose gel electrophoresis and PCR with a pair of primers in Additional file 1: Table S1 for the candidate transformants. The digestions and ligations for plasmid construction followed the methods as described by the vendor (Toyobo, Osaka, Japan).

The method for homologous recombination of strain H328 was modified, referring to that for *Thermus thermophilus* HB8 (Hashimoto et al. 2001). In brief, strain H328 was inoculated by transferring 2% (v/v) of the preculture (YS medium at 55 °C for 12 h) into the fresh YS medium (5 mL) and aerobically cultured at 55 °C until the cell growth reached OD_610_ = 0.1 to 0.15. The cells were then collected by centrifugation at 3500×*g* at 4 °C for 5 min, resuspended in fresh YS medium (0.1 mL) and cultured with 2 μg of the hybrid plasmid pUC119-*∆deg*-*htk* at 55 °C for 2 h. The cells were plated on M medium (0.05% (w/v) yeast extract, 0.15% (w/v) peptone, 0.25% (w/v) sucrose, pH 8.0) containing 100 μg kanamycin/mL and incubated at 55 °C for 72 h. The deletion of the *degP* gene in strain H328 was confirmed by PCR for the chromosomal DNA of those candidate cells with a pair of primers used for the construction of the hybrid plasmid pUC119-*∆deg*-*htk.*

### Cell growth analysis and changes in cell morphology by microscopy

Culturing was performed in YS medium as described in “Strains, plasmids, and cultivation”. Preincubations for the wild strain were carried out at 55 °C or 60 °C, corresponding to the temperature of the main culture while for the mutant strain *∆degP*, when its cell growth was measured at 55 °C, it was preincubated at 55 °C; when measured at 60 °C, it was preincubated at 57 °C for 15 h and then at 60 °C for 48 h. Cell growth was monitored for the wild and *∆degP* mutant strains by measuring the turbidity of each culture at 660 nm. The changes in cell morphology were visualized by light microscopy (Axio Imager and Axio Vision Release 4.5, Carl Zeiss, Oberkochen, Germany).

### Membrane vesicle analysis for productivity

The culture (25 mL) of strain H328 or its mutants in YS medium was withdrawn from a 2 L Erlenmeyer flask at an appropriate culture time and the supernatant was saved after centrifugation once at 5800×*g* at 4 °C for 10 min once and then twice at 20,000×*g* at 4 °C for 10 min. The saved supernatant was filtered with a PVDF membrane filter (pore size: 0.45 μm) and then ultracentrifuged at 110,000×*g* at 4 °C for 2 h (Optima L-100 K, Beckman Coulter, Brea, CA, USA). The sediments obtained by ultracentrifugation were resuspended in 500 μL of 50 mM Tris–HCl buffer (pH 8.5) and the resuspension was used as the MV fraction. To an aliquot of the MV fraction (290 μL), 150 μM 1,1’-dioctadecyl-3,3,3’,3’-tetramethylindocarbocyanine perchlorate (DiI, 10 μL) was added and incubated at 37 °C for 1 h. DiI is a lipophilic probe that is highly fluorescent when incorporated into membranes. Then the fluorescence intensity of DiI was measured at the excitation wavelength of 550 nm and the emission wavelength of 570 nm by using a fluorophotometer (RF-6000, Shimadzu, Kyoto, Japan) to determine the amounts of MVs. In the case of the amphiphilic styryl dye FM4-64, the same operation was performed to measure the fluorescence intensity at the excitation wavelength of 506 nm and the emission wavelength of 705 nm except that the incubation time with the dye was 10 min. The data were calculated as relative values to that of the wild strain at 48 h and represented with error bars as the average of two (55 °C cultivation) or three (60 °C cultivation) independent measurements. Student's *t*-tests were performed to evaluate the increase in MV production by comparing it with the value of the wild strain at 60 °C for 48 h (**, *P* < 0.01).

### Transmission electron microscopy (TEM)

The MV fractions prepared from the cultures of the wild and mutant *∆degP* strains were examined with negative staining transmission electron microscopy (TEM). Samples were adsorbed to collodion films on TEM grids (Cu, 400 mesh, Veco), sustained with 1% (w/v) phosphotungstic acid (pH7.5), and observed with TEM (Tecnai G2 F20, FEI, Hillsboro, OR, USA) operated at 120 kV.

### MALDI-TOF MS MS analysis

The samples (proteins a and b in Fig. 6) were prepared following the methods of the suppliers (Shimadzu, Kyoto, Japan) and peptide fragments extracted from SDS-PAGE were cleaved with trypsin. MS spectra were obtained with a MALDI-TOF mass spectrometer (AXIMA Resonance; Shimadzu/Kratos, Japan & UK) in positive ion mode. Protein identification was performed against a protein database of the DDBJ/EMBL/GenBank nucleotide sequence databases with accession number DF236949.2/ GAO076554.1 for strain H328.

## Results

### Gene identification of *degP* candidates in *M. ruber* H328

The homologous genes of strain H328 to the *E. coli degP* gene were searched using four different software tools—BlastP, PSORTb, CELLO, and SignalP—as described in the Materials and methods section. As a result, three genes (*mrH_0331, mrH_1124,* and *mrH_2560*) were selected as the top candidates (Table 1). Their gene products were annotated as HtrA2 peptidase, peptidase S1 and S6, and PDZ/DHR/GLGF domain-containing protein, respectively. Among the three candidates, the gene *mrH_0331* showed the highest rates of coverage (72%) and identity (36%) with the *E. coli degP* gene by BlastP analysis. Two other candidates, genes *mrH_1124* and *mrH_2560* showed lower rates of coverage and identity with the *E. coli degP* gene than with gene *mrH_0331*. Furthermore, the *E. coli degP* gene was used to annotate an *htrA2* peptidase, which is in good agreement with the annotation of gene *mrH_0331* (https://www.ncbi.nlm.nih.gov/gene/947139). Thus, gene *mrH_0331* was assigned to *degP* as a most probable candidate in strain H328.

**Table 1:** The candidate genes of *degP* in the genome of strain H328 by use of four different types of softwares

Gene number in the strain H328 genome Gene name by annotation (amino acid number in the open reading frame, molecular weight (kDa))	Homology (coverage (%) / identity (%) *vs. E. coli degP*)	Localization^a^		Signal sequence^b^
	BlastP^c^	PSORTb^d^	CELLO^e^	SignalP 4.0^f^
*mrH_0331* HtrA2 peptidase (413, 44.5)	72% / 36%	P	IM, P, OM	△
*mrH_1124* Peptidase S1 and S6 (344, 37.6)	69% / 25%	P	IM	△
*mrH_2560* PDZ/DHR/GLGF domain-containing protein (352, 37.2)	62% / 29%	P	IM, OM	○

^a^IM, inner membrane; P, periplasmic; OM, outer membrane

^b^○, high possibility; △, moderate possibility

^c^http://blast.ncbi.nlm.nih.gov/Blast.cgi

^d^http://www.psort.org/psortb/

^e^http://cello.life.nctu.edu.tw/

^f^http://www.cbs.dtu.dk/services/SignalP-4.0/

### Homologous recombination to obtain the *degP*-deleting mutant

To obtain the mutant, the hybrid plasmid for the homologous recombination of strain H328 was constructed (Fig. 1). DNA fragments including the 5’- and 3’-flanking regions of the *degP* gene (*mrH_0331*) were prepared by PCR by using the respective pair of primers (*degP*/F1-Hind and *degP*/R2-Pst for the 5’-flanking region and *degP*/R3-Pst and *degP*/R4-Xba for the 3’-flanking region) listed in Additional file 1: Table S1 and were inserted into vector plasmid pUC119 at the *Hind*III and *Xba*I sites after digesting the DNA fragments with *Hind*III/*Pst*I and *Pst*I/ *Xba*I, respectively. The resulting plasmid was named pUC119-Up-Down-*degP*. The plasmid was ligated with the DNA fragment containing the *htk* gene and its promoter for thermophilic kanamaycin resistance to construct the hybrid plasmid pUC119-*∆degP*-*htk* (Fig. 1A). Plasmid pUC119-*∆degP*-*htk* was employed to achieve homologous recombination at the *degP* gene in *M. ruber* H328. The method for homologous recombination was performed as described in the Materials and methods section. When 2 μg of the plasmid was used for homologous recombination with about 10^8^ cells per the plate of M medium containing 100 μg kanamycin/mL, colonies showing kanamaycin resistance appeared with an efficiency of 2.5 × 10^–5^ on the plate at 55 °C in 72 h. For one of the representative *∆degP* mutant strains obtained, PCR was performed, and a DNA fragment of the same size as the structural gene of *degP* was discovered to be linked to the 5'-flanking and 3'-flanking regions of the *degP* gene (lanes 1 and 2 in Additional file 1: Fig. S1).The deletion of the *degP* gene was also confirmed by PCR for the *∆degP* mutant strain with a pair of primers (*degP*/ORF/Fw and *degP*/ORF/Rv, Additional file 1: Table S1, lanes 3 and 4 in Additional file 1: Fig. S1) while the replacement by the *htk* gene was observed by PCR with a pair of primers (*htk*/Fw-Pst and *htk*/Rv-Pst, Additional file 1: Table S1, lane 6 in Additional file 1: Fig. S1). Furthermore, the orientation of the *htk* gene was identified by PCR with a pair of primers (*degP*/F1-Hind and *htk*/Rv-Pst or *htk*/Fw-Pst and *degP*/R4-Xba, data not shown). As a result, the mutant strain *∆degP* was found to include the desired homologous recombination at the *degP* gene.

### Cell growth analysis and changes in cell morphology

**Fig. 2:**
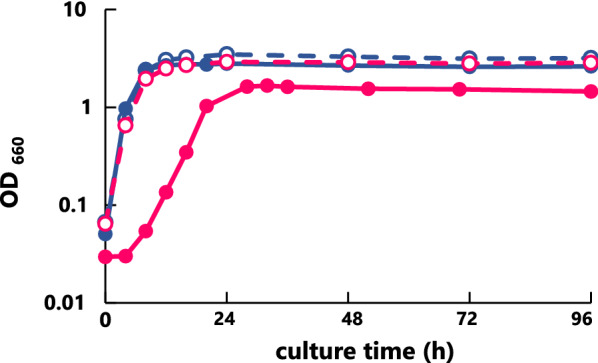
Cell growth of the wild and *∆degP* mutant strains in YS medium. The strains were incubated at 55 °C (wild strain, ; *∆degP* strain, ) or 60 °C (wild strain, ; *∆degP* strain, ) in YS medium after preincubation at various temperatures. Preincubation was carried out at 55 °C for the wild strain. As for the *∆degP* mutant strain, when its cell growth was measured, it was preincubated at 55 °C; when measured at 60 °C, it was preincubated at 57 °C for 15 h and then at 60 °C for 48 h. The cell growth of each strain was monitored by measuring its turbidity at 660 nm for 96 h

The cell growth of the wild and mutant strains (H328 and *∆degP*) was investigated (Fig. 2). The wild strain showed a similar pattern of cell growth in cultivations at 55 °C and 60 °C. The mutant strain *∆degP* also exhibited comparable growth at 55 °C. In contrast, preliminary investigations of mutant strain *∆degP* showed that growth was arrested at 60 °C after preculture at 55 °C overnight and it was necessary to habituate to the 60 °C temperature during preculture (data not shown). Based on this finding, we changed the preculture temperature to investigate the growth of the mutant strain at 60 °C. As a result, the preculture was carried out at 57 °C for the first 15 h and then for 48 h at 60 °C in YS medium, followed by incubation at 60 °C, in order to investigate the cell growth of the mutant strain *∆degP* (Fig. 2). The cell grown of the mutant strain was smooth but at the lower level of turbidity at 60 °C. This indicates that the cell growth of the mutant strain was impaired due to heat stress by incubation at 60 °C and that the impairment was caused by the deletion of the *degP* gene.

**Fig. 3:**
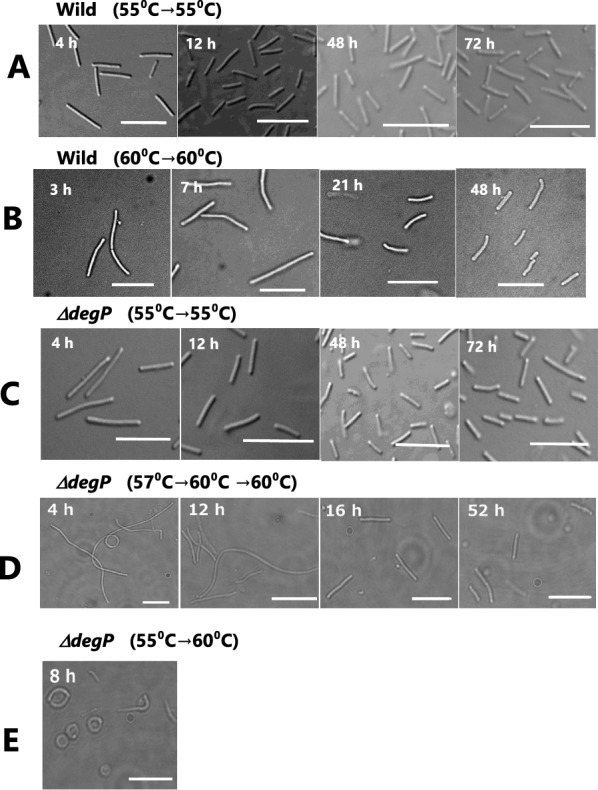
Changes in cell morphology of the wild and *∆degP* mutant strains along the time course at 55 or 60 °C cultivation. The cultivation was carried out at 55 or 60 °C after preincubation at various temperatures; **A**, the wild strain of H328 at 55 °C for 4, 12, 48, and 72 h cultivation after preincubation at 55 °C overnight; **B**, the wild strain of H328 at 60 °C for 8, 20, 52, and 96 h cultivation after preincubation at 60 °C overnight; **C**, *∆degP* mutant strain at 55 °C for 4, 12, 48, and 72 h cultivation after preincubation at 55 °C overnight; **D**, *∆degP* mutant strain at 60 °C for 4, 12, 16, and 52 h cultivation after preincubation for 15 h at 57 °C and then 48 h at 60 °C; **E**, *∆degP* mutant strain at 60 °C for 8 h cultivation after preincubation at 55 °C overnight. The scale bars are 10 μm in length

Changes in cell morphology were traced by light microscopy along the time course of cell growth at 55 °C and 60 °C after preincubation at various temperatures (Fig. 3). The mutant strain *∆degP* (Fig. 3C) exhibited the same rod-like morphology as the wild strain at 55 °C (Fig. 3A, B); however, it arrested growth and showed a different morphology at 60 °C after preculture overnight at 55 °C. The cell morphology of the mutant strain *∆degP* was not rod-shaped but round with the cells attached at both edges at 60 °C after incubation in 8 h (Fig. 3E). In contrast, preculture for the mutant strain at 57 °C and 60 °C followed by incubation at 60 °C resulted in longer rods at 4 h, but short rods at 16 h, which is a result similar to that of the wild strain (Fig. 3D). However, it should be noted that the mutant strain *∆degP* showed significant retardation in growth and reduced turbidity (Fig. 2).

### Enhancement of MV production revealed by employing fluorescent reagents DiI and FM4-64

**Fig. 4:**
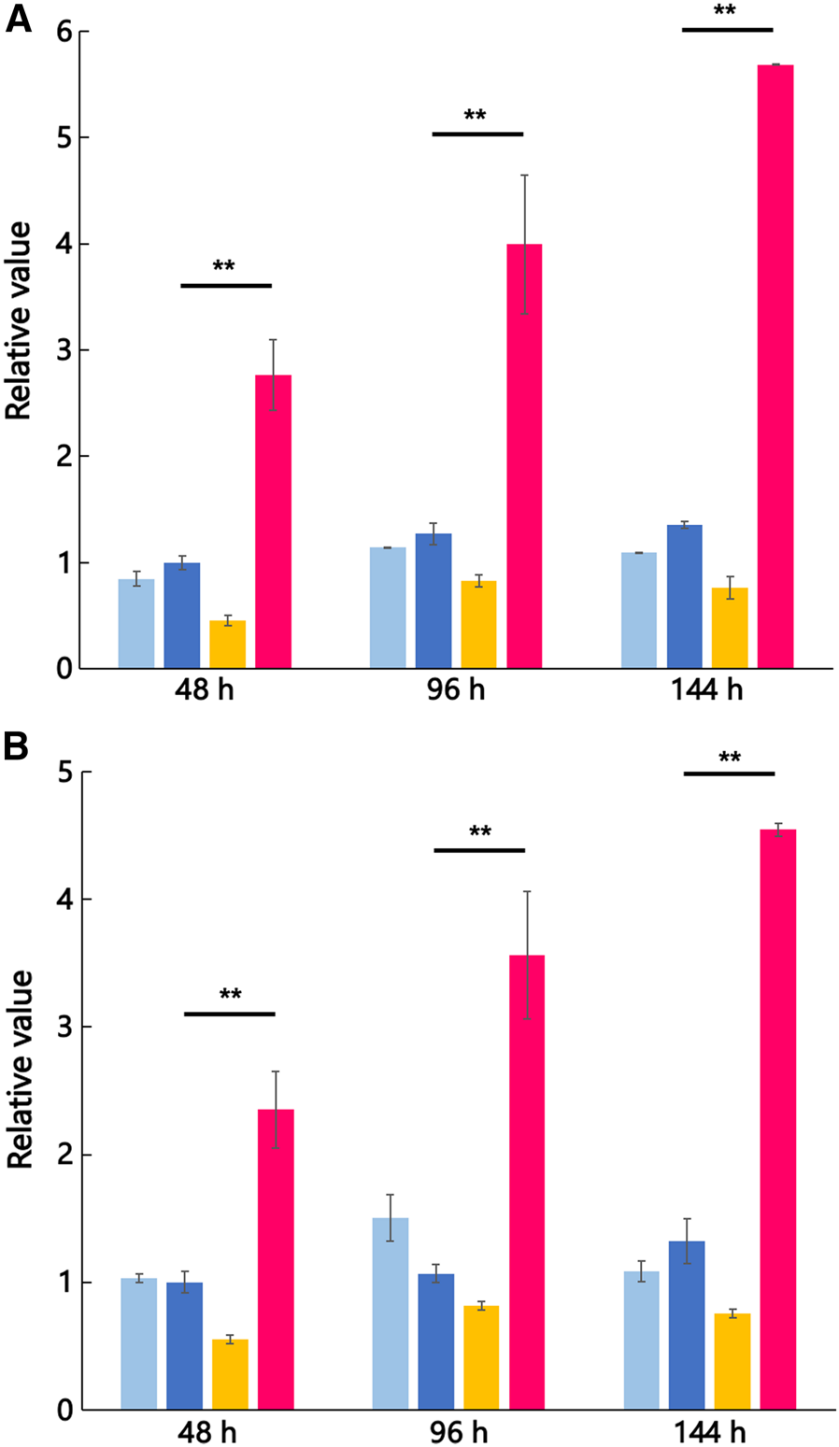
Comparison of MV production between wild and mutant strains at 55 °C or 60 °C with different cultivation times (48, 96, and 144 h) using fluorescent reagent DiI (**A**) or FM4-64 (**B**). Culture supernatants were obtained from the culture incubating at 55 °C or 60 °C at different times (h) and the samples were prepared by following the methods as described in the Materials and methods section. The data were calculated as values relative to that of the wild strain at 48 h and are represented by error bars as the average of two (55 °C cultivation) or three (60 °C cultivation) independent measurements. Student's *t*-tests were used to evaluate the increase in MV production as compared with the value of the wild strain at 60 °C for 48 h (**, *P* < 0.01). **A** and **B**, the wild strain of H328 at 55 °C after preincubation at 55 °C overnight (); the wild strain of H328 at 60 °C after preincubation at 60 °C overnight (); *∆degP* mutant strain at 55 °C after preincubation at 55 °C overnight (); *∆degP* mutant strain at 60 °C after preincubation for 15 h at 57 °C and then 48 h at 60 °C ()

The MV production of mutant strain *∆degP* was examined by using two fluorescent reagents DiI and FM4-64. The fluorescent reagents have been employed previously to measure cell membranes by penetrating the lipid bilayer membrane with hydrophobic side chains (Honig and Hume 1986, 1989) and MV concentrations (McBroom et al. 2006; Macdonald and Kuehn 2013). As seen in Fig. 4 A and B, the mutant strain incubated at 60 °C was found to be able to produce more MV than the wild strain at 48, 96, and 144 h. The degree of enhancement increased with incubation time (48, 96, and 144 h) and was found to be about five times higher than that of the wild strain at 144 h of cultivation. This implies that the rapid MV purification method using the ultracentrifugation system for MV preparation is fully significant and that the staining of MVs with the two fluorescent dyes DiI and FM4-64 was similarly performed. As a result, the increase in MV at 60 °C was the same. Moreover, it should be noted that the increase was not observed for the mutant strain *∆degP* incubated at 55 °C. The difference suggests that strain H328 had different temperature sensitivities at 55 °C and 60 °C, corresponding well to the fact that the growth of the mutant strain was impaired at 60 °C (Fig. 3).

### TEM observation

**Fig. 5:**
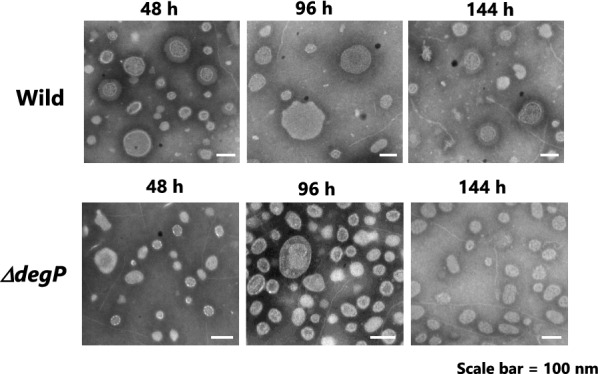
TEM analysis for MV fractions prepared from the cultures of the wild and mutant strains with different cultivation times (48, 96, and 144 h). The samples were prepared from the culture supernatants according to the method described in the Materials and methods section. The scale bars are 100 nm in length

The morphologies of MVs were examined with negative staining TEM in MV fractions prepared from the cultures of the wild and mutant strains in different cultivation times (48, 96, and 144 h) at 60 °C (Fig. 5). In the fractions of the wild strain, MVs of the regular size (100–400 nm wide; Yamaoka et al. 2014) were observed along the time course, whereas in the MV fractions of the mutant strain *∆degP*, the majority of MVs appeared smaller than 100 nm*.* However, since MVs were concentrated upon the adsorption on the surface of TEM grids, the density of MVs in electron micrographs did not quantitatively reflect that in the sample solutions. It was difficult to verify the increase in the number of MVs from TEM images.

### Increase of SlpA in the MV fraction

**Fig. 6:**
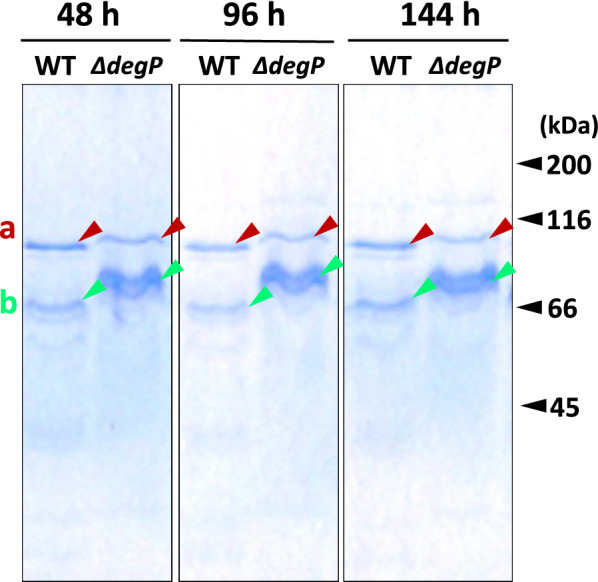
SDS-PAGE profiles for the MV fractions prepared from the culture at 44, 96, and 144 h incubation. WT: from the wild strain in the respective culture time, *∆degP*: from the mutant strain *∆degP* in the respective culture time. The same amount (20 μL) of the MV fraction was applied for each lane. The gel concentration was 10.5% (w/v) acrylamide. Two main bands are depicted with red arrows for protein a and green arrows for protein b

Proteins in MV fractions prepared from the wild and mutant strains grown at 60 °C were examined by SDS-PAGE. As shown in Fig. 6, it was found that only a limited number of proteins, in particular, one band of protein a and a few bands of protein b, occurred in the profile. A few bands of protein b appeared, but they were identified as the same protein, as will be shown later. On the other hand, this pattern was very similar to that of the peak 1 fraction prepared with Sephacryl S-1000 (exclusion limit: 10^8^ Da) (Kataoka et al. 2014). Those two proteins were subjected to MALDI-TOF MS MS analysis and both were identified as S-layer protein (*mrH_2961*) which we named SlpA. Software PSORTb found that the S-layer protein localized in the outer membrane (see Table 1). This result is in good agreement with the fact that MVs are composed of an outer membrane and accumulate in this MV fraction prepared by the method as described. Furthermore, it should be noted that, as measured by the software Image J software (https://imagej.nih.gov/ij/index.html), the quantity of protein a did not change much, whereas protein b was found to be four times higher in the mutant strain than in the wild strain after incubation at 60 °C for 144 h (data not shown). This indicates that SlpA was concentrated in the MV fraction.

## Discussion

It has been reported that all bacteria release membrane vesicles (MVs) regardless of whether they are Gram-negative or -positive bacteria (Mayer and Gottschalk 2003). Since an increasing number of reports have been published, more attempts to apply MVs have been made in various directions, including the medical fields (Liu et al. 2018; Martens-Uzunova et al. 2021; Morishita et al. 2021; Pillalamarri et al. 2021). This background will expand the potential of MV applications if more MVs can be produced by bacteria.

In our study, we focused on the *degP* gene, which, in *E. coli,* has been demonstrated (Schwechheimer and Kuehn 2013) to improve the MV production capacity in *Meiothermus ruber* H328. We first selected three genes of candidates (*mrH_0331, mrH_1124,* and *mrH_2560*) for the *degP* gene from the H328 genome and, secondly from the analyses of homology, localization, and gene expression, concluded that gene *mrH_0331* was assigned to *degP* as a most probable candidate in strain H328 (Table 1). We then constructed the mutant strain *∆degP* deleting the *degP* gene in strain H328, which was replaced by the *htk* gene showing thermophilic kanamaycin resistance by homologous recombination (Fig. 1). The mutant strain *∆degP* exhibited smooth growth but a lower level of turbidity at 60 °C although there was no difference in growth at 55 °C between the wild strain and the mutant strain of *∆degP* (Fig. 2). In addition, preincubation at 57 °C and 60 °C was indispensable for the mutant strain *∆degP* to adapt itself to the 60 °C incubation.

From this finding, we assume that the cell growth of the mutant strain would be impaired at 60 °C due to heat stress by incubation and that this impairment would be caused by the deletion of the *degP* gene. Originally, DegP protein is a heat shock protein, which is a periplasm-localized chaperone/serine protease (Lipinska et al. 1990). Therefore, the deletion of the *degP* gene conferred more susceptibility to the growth of strain H328 at 60 °C. As proof, the cell morphology of the mutant strain *∆degP* incubating at 60 °C followed by preincubation at 55 °C definitely changed from rod-shape to rounded shape at both edges of cells (Fig. 3). This change could be caused by increasing heat-denatured proteins by *degP* knockout. In separate experiments, by using MALDI-TOF MS MS analysis, we found that the S-layer protein included in the outer membrane of the H328 strain increased at 60 °C (Fig. 6). Since the denatured protein was not identified in the *degP* deletion mutant of *E. coli* (Schwechheimer and Kuehn 2013), it is not yet known what the denatured protein in the mutant strain *∆degP* is; however, the S-layer protein is one of the candidates of those proteins. Finally, we have confirmed that incubation at 60 °C increases MV production in the mutant strain *∆degP* up to about fivefold by the use of two fluorescent dyes DiI and FM4-64 (Fig. 4), which is followed by TEM analysis (Fig. 5). This indicates that the MV production did not increase in the wild and mutant strains at 55 °C because they did not accumulate denatured proteins due to heat shock, whereas the mutant strain only enhanced MV production at 60 °C because it accumulated denatured proteins. The difference in MV production by strain H328 at these two temperatures occurs well in the growth of the wild and mutant strains, indicating that 60 °C is a heat-stressing condition in the growth of the H328 strain. In addition, the method with florescent dyes employed to detect MVs from culture media is suitable for measuring MV accumulation due to the intensive concentration of SlpA protein and can be done in several hours, even if including ultracentrifugation and filtration for cell exclusion, which is much shorter than the method used thus far.

Furthermore, since two other candidates, *mrH_1124 and mrH_2560* showed similar results in spite of lower homology (Table 1), they should be good candidates for the *degP* gene. Therefore, we are continuing to investigate them.

Genetic manipulations other than of the *degP* gene have been reported to increase MV production capacity (Table 2). These genes are neither directly related to the *degP* gene nor other heat-stress genes, which implies that the increase in heat-denatured proteins does not necessarily lead to an enhancement of MV production. It should also be noted that these genes cannot be annotated in the genome of strain H328, i.e. it is highly unlikely that the homologues exist in strain H328. In this context, this enhancement in MV production by the disruption of the *degP* gene may be an example that can be applied to other thermophilic bacteria. In addition, it must be taken into account that MV biogenesis has not yet been fully established. It has been suggested that they are foliated from the cell surface (Kulp and Kuehn 2010), that they are formed during cell rupture (Turnbull et al. 2016), or that they are the result of the aggregation of components in parts of the cell membrane (Roier et al. 2016). It is more likely that further reports of novel gene disruptions leading to enhanced MV production and MV biogenesis will be clarified in the future.

**Table 2:** Summary of genes that contribute to improved OMV production in bacteria

Gene	Function	*Meiothermus ruber*	*Escherichia coli*	*Salmonella enterica*	*Helicobacter pyroli*	*Pseudomonas aeruginosa*	References
*degP*	Periplasmic serine protease	○	○	○	○	×	Schwechheimer and Kuehn (2013)
*tolRA*	OM integrity	×	○	○	○	○	Turner et al. (2015) Bernadac et al. (1998) Daleke-Schermerhorn et al. (2014)
*nlpI*	OM lipoprotein	×	○	○	×	×	McBroom et al. (2006) Nevermann et al. (2019)
*gmhB*	D-glycero-β-D-manno-heptose-1,7-bisphosphate 7-phosphatase	×	○	○	○	○	Kulp et al. (2015)
*rfaD*	ADP-L-glycero-D-mannoheptose 6-epimerase	×	○	○	○	○	Kulp et al. (2015)
*rfaE*	Heptose 7-phosphate kinase/heptose 1-phosphate adenyltransferase	×	○	○	○	○	Nevermann et al. (2019)
*vacJ/yrbB*	ABC transport system IM phospholipid transport system protein	×	○	○	×	×	Roier et al. (2016)

*OM* outer membrane, *IM* inner membrane

In conclusion, various ideas for MV biogenesis have been proposed, but no definitive mechanism has yet been established. The deletion of the *degP* gene in strain H328 effectively enhances MV production. Although a rigid relationship between the production of MVs and feather degradation has not yet been confirmed, we are investigating the potential of the wild and mutant strains (H328 and *∆degP*) to degrade chicken feathers at 60 °C under heat stress greater than 55 °C.

## Supplementary Information

**Additional file 1: Fig. S1.** PCR for the mutant strain *∆degP* to examine desired homologous recombination at the locus of *degP* gene. Lane M, kb-ladder; lane 1, PCR for the genome DNA of the wild type strain *M. ruber* H328 with primers (*degP*/F1-Hind and *degP*/R4-Xba); lane 2, PCR for the genome DNA of the mutant strain *∆degP* with the same primers; lane 3, PCR for the genome DNA of the wild type strain with primers (*degP*/ORF/Fw and *degP*/ORF/Rv); lane 4, PCR for the genome DNA of the mutant strain *∆degP* with the same primers; lane 5, PCR for the genome DNA of the wild type strain with primers (*htk*/Fw-Pst and *htk*/Rv-Pst); lane 6, PCR for the genome DNA of the mutant strain *∆degP* with the same primers. **Table S1.** DNA primers used for plasmid construction and screening of *degP* gene in strain H328.

## Data Availability

All data are incorporated into the article and its online Additional file.
