# Supplementary material for: Enhancement of membrane vesicle production by disrupting the degP gene in Meiothermus ruber H328
Source: AMB Express. 2021 Dec 15;11:170. doi: 10.1186/s13568-021-01328-z (PMC8674399; doi:10.1186/s13568-021-01328-z)
Supplement: Supplementary file 1 — Additional file 1: Fig. S1. PCR for the mutant strain ∆degP to examine desired homologous recombination at the locus of degP gene. Lane M, kb-ladder; lane 1, PCR for the genome DNA of the wild type strain M. ruber H328 with primers (degP/F1-Hind and degP/R4-Xba); lane 2, PCR for the genome DNA of the mutant strain ∆degP with the same primers; lane 3, PCR for the genome DNA of the wild type strain with primers (degP/ORF/Fw and degP/ORF/Rv); lane 4, PCR for the genome DNA of the mutant strain ∆degP with the same primers; lane 5, PCR for the genome DNA of the wild type strain with primers (htk/Fw-Pst and htk/Rv-Pst); lane 6, PCR for the genome DNA of the mutant strain ∆degP with the same primers. Table S1. DNA primers used for plasmid construction and screening of degP gene in strain H328. [file 13568_2021_1328_MOESM1_ESM.docx]

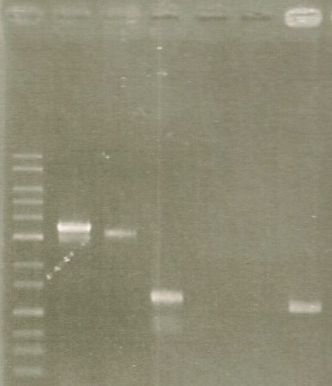


**M**

**1**

**2**

**3**

**4**

**5**

**6**

**(kb)**

**3**

**2**

**1**

Fig. S1. PCR for the mutant strain *ΔdegP* to examine desired homologous recombination at the locus of *degP* gene. Lane M, kb-ladder; lane 1, PCR for the genome DNA of the wild type strain *M. ruber* H328 with primers (*degP*/F1-Hind and *degP*/R4-Xba); lane 2, PCR for the genome DNA of the mutant strain *ΔdegP* with the same primers; lane 3, PCR for the genome DNA of the wild type strain with primers (*degP*/ORF/Fw and *degP*/ORF/Rv); lane 4, PCR for the genome DNA of the mutant strain *ΔdegP* with the same primers; lane 5, PCR for the genome DNA of the wild type strain with primers (*htk*/Fw-Pst and *htk*/Rv-Pst); lane 6, PCR for the genome DNA of the mutant strain *ΔdegP* with the same primers.

Fig. S1 Asano et al.

Table S1. DNA primers used for plasmid construction and screening of *degP* gene in the strain H328.

| name | Sequence (5’ → 3’) | Description or reference |
| --- | --- | --- |
| *degP*/F1-Hind | CCCAAGCTTACCTGTGGCACAGGTGGGTT | Construction of 5’-flanking region (1.0 kbp) of *degP* gene as forward primer |
| *degP*/R2-Pst | GAACTGCAGCTCAAAGACCTCCACCTAGC | Construction of 5’-flanking region (1.0 kbp) of *degP* gene as reverse primer |
| *degP*/F3-Pst | GAACTGCAGGAGGGTTTTCCTTTGCTTC | Construction of 3’-flanking region (1.0 kbp) of *degP* gene as forward primer |
| *degP*/R4-Xba | TGCTCTAGACTCATTGACGATGATACGCA | Construction of 3’-flanking region (1.0 kbp) of *degP* gene as reverse primer |
| *htk*/Fw-Pst | GAACTGCAGCGTTGACGGCGGATATGGTA | Construction of the open reading frame of *htk* gene as forward primer |
| *htk*/Rv-Pst | GAACTGCAGCGTAACCAACATGATTAACA | Construction of the open reading frame of *htk* gene as reverse primer |
| *degP*/ORF/Fw | ATGTCCATGAGAAATTCTTC | Construction of the open reading frame of *degP* gene as forward primer |
| *degP*/ORF/Rv | TTAGCGGATCACCTGCGGCA | Construction of the open reading frame of *degP* gene as reverse primer |
